# Nanoscopic live electrooptic imaging

**DOI:** 10.1038/s41598-021-85095-8

**Published:** 2021-03-10

**Authors:** Masahiro Tsuchiya, Shigeru Takata, Kazuhiro Ohsone, Shinji Fukui, Muneo Yorinaga

**Affiliations:** 1 National Institute of Information and Communications Technology, 4-2-1 Nukui-Kitamachi, Koganei-shi, Tokyo, 184-8795 Japan; 2 Nippon Soken Inc., 14 Iwaya, Hasumi-cho, Nishio-shi, Aichi, 445-0012 Japan

**Keywords:** Nanoscience and technology, Optics and photonics

## Abstract

A door to the nanoscopic domain is opened regarding real-time visualization of electric field distributions and dynamics. Through the use of a live electrooptic imaging system with an oil-immersion objective lens and a highly thinned electrooptic sensor film, a minimum linewidth of 330 nm and a minimum peak splitting of 650 nm in real-time electric field video images have been successfully demonstrated. In addition, room to improve the resolution is noted, while a few problems that need to be solved are discussed, including an effect caused by optical interference.

## Introduction

**Figure 1:**
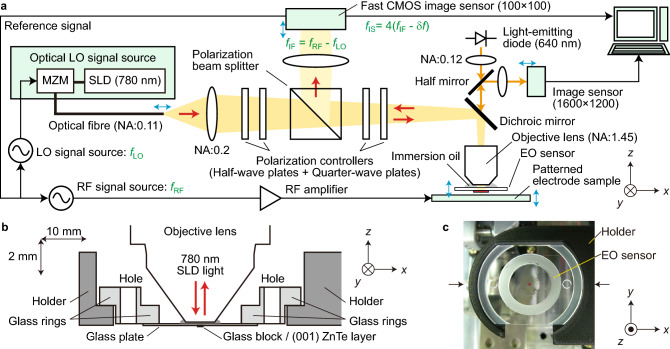
LEI system. The optics include an oil-immersion objective lens and a thin film EO sensor. (**a**) Schematic drawing of the present LEI system. Blue arrows indicate fine positioners. Frequencies *f*_RF_, *f*_LO_, *f*_IF_, *f*_IS_, and δ*f* are explained in the main text and the “Methods” section. (**b**) Centrally sectioned drawing of the oil-immersion objective lens and EO sensor. The *x* and *z* directions have anisotropic magnifications. (**c**) Top-view photograph of the EO sensor set in its holder. *CMOS* complementary metal–oxide–semiconductor, *IF* intermediate frequency, *LO* local oscillator, *MZM* Mach Zehnder optical modulator, *NA* numerical aperture, *RF* radio frequency, *SLD* super-luminescent diode.

A prompt understanding of the spatial domain behaviour of invisible electrical phenomena is highly attractive for many fields in science and technology. Real-time visualization of electric fields in terms of their spatial distribution and high-speed dynamics is ideal for this purpose. A technique named live electrooptic (EO) imaging (LEI)^1–3^, whose present configuration is shown in Fig. 1, has provided a unique solution with the advantage of experimental agility; two-dimensional (2D) electric field distributions up to the GHz range are visualized in real-time phase-evolving video formats. Regarding the spatial resolution, which is one of the most important LEI characteristics, the conventional highest resolution is 2.8 μm^4^, and its extension to the nanometric range, where various fundamental phenomena occur electrically, is quite meaningful from scientific and technological points of view.

In this paper, we open the door to nanometric resolution for the LEI technique.

Real-time electric field images acquired in this work indicate (a) a minimum linewidth of 330 nm and a minimum peak splitting of 650 nm with more signal to noise ratios than 20 dB, (b) room for further improvement of the resolution, and (c) a few problems to be solved, including an effect caused by optical interference.

The LEI technique is based on two advantages of photonics^5^, the ultra-parallel^6,7^ and ultra-fast^8,9^ properties, which are merged in an EO crystal^10,11^ shaped into a thin layer^12–14^. In the EO layer, a key role is performed by the linear EO effect known as the Pockels effect: the optical refractive index of a crystalline solid changes linearly and instantaneously upon application of an electric field to the crystal. In an LEI system, an electric field distribution at a radio frequency (RF) *f*_RF_ induces a 2D change in the refractive index of an EO layer, which is transferred to a cross-sectional phase modulation distribution in the beam of an optical local oscillator signal (OLOS) modulated originally at a local oscillator (LO) frequency *f*_LO_. Then, an ultra-parallel frequency down-conversion process occurs in the polarization optical system together with a modulation format conversion. The 2D distribution of the intensity modulation thus generated at an intermediate frequency (IF) *f*_IF_ ($={f}_{\mathrm{RF}}-{f}_{\mathrm{LO}}$) in the kHz range (typically 5 kHz) is photodetected in quadrature by a high-speed complementary metal–oxide–semiconductor (CMOS) image sensor in an ultra-parallel manner (typically 100 × 100), leading to display of real-time phase-evolving electric field videos at a display frame rate *f*_D_ (typically 10 frames per second). The 2D distributions of the amplitude and phase of the original RF signal are evaluated through the IF signal via quadrature photodetection by each pixel of the CMOS image sensor and subsequent signal processing, including averaging by a factor of *f*_IF_/*f*_D_ for noise reduction. The absence of mechanical scans in the LEI technique is potentially beneficial for a massive number of pixels in future real-time EO imaging because of the parallel signal processing embedded in an image sensor and its interface circuitry^15^. Simultaneously, electric field sources, such as a patterned electrode sample, beneath the EO sensor are optically monitored in a dichroic manner by a slow (standard) image sensor device using illumination provided by a light-emitting diode (LED). Detailed information on other LEI generalities is provided in the literature^1–3^ as well as in the “Methods” section.

### Setup

Distinct in this work are the following three factors. First, an optical system of nanometre-scale resolution has been constructed that is compatible with the following thin EO crystal layers, which are called EO films hereafter. Second, EO films of nanometre thicknesses have been prepared for improved resolution as well as improved accessibility to the following nanometre-scale electric field distributions. Third, fine electrode patterns have been fabricated to generate electric field distributions that spatially vary on the nanometre scale. Their details are described below.

The LEI polarization optics shown in Fig. 1a contain an oil-immersion objective lens (Olympus UAPON 150XOTIRF) with a magnification of 150 diameters and an aberration compensation mechanism for a glass thickness range of 130–190 μm. Its numerical aperture (NA) is 1.45, which is almost triple the conventional value (0.5)^4^, leading to a Rayleigh criterion^16^ of 328 nm and an Abbe diffraction limit^16^ of 538 nm. Its Hopkins’ resolution^16^ is calculated to be 441 nm, taking the NA value (0.11) of the OLOS condenser into account. These optical resolution numbers are more than double the pixel pitch (145 nm) and are therefore the basis of the EO image resolution. Here, the depth of the optical focus is 460 nm^16^.

As shown in Fig. 1b, which presents a drawing of the central section of the oil-immersion objective lens and EO sensor with anisotropic magnifications in the *x* and *z* directions, a typical upright immersion microscope system is configured downward from the objective lens to the focal plane, i.e., an EO film, with immersion oil (80 μm), a glass plate (100 μm), and a glass block (60 μm) in between. The total thickness of the glass components is within the range of aberration compensation provided by the objective lens. To the best of our knowledge, such an oil-immersion system has never been applied to EO sensing. The glass plate is adhered to a glass ring structure for attaching/detaching an EO sensor to/from its holder closely designed for the oil-immersion system, as explained in the “Methods” section. The top-view image in Fig. 1c includes an EO sensor set in the holder with the objective lens and patterned electrode sample removed. The holder is bolted to a 5-axis stage on the right-hand side of the image, which has *x*–*y*–*z* positioners with submicron precision and in-plane tilt angle adjusters. In the photograph, some residue of the immersion oil is noticeable on the glass plate together with a small red square at its centre, which is a (001) ZnTe thin film sensitive to electric fields in the *z* direction ${E}_{z}\left(x,y\right)$.

The OLOS source consists of a super-luminescent diode (SLD) and a Mach Zehnder optical intensity modulator (MZM), both of which are for the 780 nm wavelength. The wavelength choice of 780 nm within the sensitive range of the CMOS image sensor originates from the MZM availability. The OLOS coherent length *L*_c_ is estimated to be 18 μm from its measured spectra. This short coherence length property gives rise to suppression of the unnecessary interference pattern effect^17^ but was found to not be sufficient, as described later.

**Figure 2:**
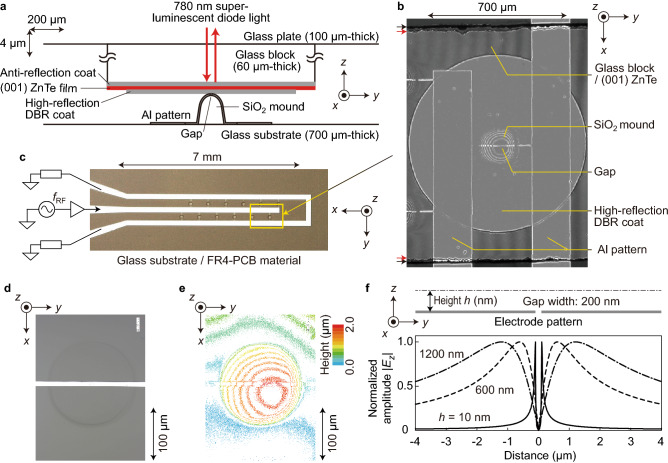
EO sensor on a nanometre-scale electrode pattern. (**a**) Cross-sectional schematic of a thin ZnTe film on a gap of an electrode pattern formed over a SiO_2_ mound. Here, the anisotropic magnification ratio of the *y-*direction to the *z*-direction is 1:40. (**b**) Top-view photograph of the same setup taken at a low magnification. Red and black arrows indicate the edges of the glass block and ZnTe film, respectively. (**c**) Top-view photograph of an electrode pattern on a 700-μm-thick glass substrate adhered to an FR4 material. Additionally, external driving and termination circuits are schematically indicated. (**d**) Magnified optical image of one of the SiO_2_ mounds taken by a confocal laser microscope without the EO sensor. (**e**) Three-dimensional structure of the mound in (**d**). (**f**) Theoretical model for calculation of the *E*_*z*_ amplitude distribution above an electrode pattern with a 200-nm-wide gap along with the results calculated at three values of the height *h*. DBR: distributed Bragg reflector.

The EO sensor shown in Fig. 2a is distinguished by two highly thin films: the ZnTe crystal film and its bottom coating of a distributed Bragg reflector (DBR) stack for high reflection (HR). Generally, the distributions of the electric fields that originate from in-plane electric charges and stretch upward are finest at the pattern surface, disperse sideways when moving away from the charges, and superpose with adjacent electric fields in the upper space. To realize the ultimate LEI spatial resolution, *E*_*z*_ distributions should therefore be imaged as near as possible to the nanometre-scale charge distributions. Ideally, a zero-thickness EO film with a zero-thickness HR coating would touch a nanometre-scale charge distribution. However, their real thicknesses are finite, in which the LEI spatial resolution is degraded by the EO effect vertically integrated through the ZnTe film and by the low proximity caused by the HR coating.

Nevertheless, the thickness of the EO film was reduced from 1.5 μm to 560 nm in comparison with the conventional film^4^, and the DBR thickness was reduced from 1.4 μm (6 pairs of high and low refractive index layers) to 230 nm (1 pair). Simultaneously, the reflectance of the single-pair DBR became almost half the conventional value (> 90%), 53% at the OLOS wavelength (780 nm), leading to a nonnegligible amount of light transmission. As discussed more intensively later, transmitted light is reflected at a patterned electrode surface and meets DBR-reflected light. Most of their optical path differences are in the submicron range and less than *L*_c_. Consequently, interference patterns are generated, accompanied by their effects on electric field images. This effect is related to the trade-off with the DBR thickness, i.e., the EO sensor proximity.

SiO_2_ mound structures, on which nanometre-scale gap electrode structures were formed, as shown in Fig. 2a, were uniquely employed to avoid obstructions caused by dust particles; the flat EO film would suffer from its degraded proximity to a standard flat electrode pattern induced by a small number of dust particles in between. The mound structure reduces the probability of dust obstruction in proportion to the area ratio of the mound tops to the other regions, although the area for electric field imaging is highly restricted by the ratio. In addition, the characteristic impedances of the patterns are modified, and the frequency characteristics of the signal transmission are affected. Figure 2a shows a cross-sectional schematic of the Al pattern on a mound and an EO sensor, where the anisotropic magnification ratio of the *y-*direction to the *z*-direction is 1:40. The mound structures were deposited on a 700-μm-thick glass substrate via RF sputtering using a 200-μm-thick metal mask. Their diameters and peak heights are approximately 100 μm and 2–5 μm, respectively. The Al line patterns, whose designed width is 10 μm, were formed over the mounds by RF sputtering, photolithography and wet etching.

Figure 2b shows a cutout of a photograph taken using an objective lens of low magnification (× 5), 640 nm LED illumination with a collimating lens of NA = 0.12, and a standard CMOS image sensor with 1600 × 1200 pixels, where the ZnTe film was set close to a mound top. The photograph includes two 200-μm-wide principal electrodes along the *x* direction and the abovementioned narrow line electrodes in between. A set of Newton’s rings appears at the middle of the narrow electrode, which is created by the mound structure and the HR coating of the ZnTe film, suggesting their proximity as well as the shape of the mound. Additionally, a nanometre-scale gap is visible at the centre of the Newton’s rings. In addition, Fig. 2b includes the disk-shaped HR coating and *x*-direction edges of the ZnTe film and glass block, which are indicated by red and black arrows, respectively. The Al pattern and the EO sensor are not necessarily in contact, and the field of view can be shifted under focused conditions if their distance is kept unchanged in the low nanometric range.

The photograph in Fig. 2c shows an extended area of the electrode pattern, which was taken using a standard microscope, together with a schematic of the driving and termination circuitries. The glass substrate on which Al patterned electrodes and SiO_2_ mounds were formed was adhered on a board of a flame-retardant type 4 (FR4) material. An RF signal at *f*_RF_, which is generated by an RF synthesizer and is injected into the central principal electrode, passes through the gaps on the narrow line electrodes, reaches the outer principal electrodes, and terminates at the 50-Ω loads. A patterned electrode sample includes 10 narrow line electrodes and mounds.

A magnified image of one of the SiO_2_ mound structures was taken by a confocal laser microscope (Keyence VK-9510) without the EO sensor and is shown in Fig. 2d together with its height map in Fig. 2e. The tilt of the pattern surface around the mound top was measured to be 100 nm/8 μm. The electric field distribution to be observed is succinctly predicted by an electromagnetic conformal mapping method, in which the thickness of the Al pattern is assumed to be zero and the gap width 200 nm, as shown in the upper part of Fig. 2f. The calculated distributions of the *E*_*z*_ amplitude are shown in the lower part of Fig. 2f, with the distance *h* from the Al pattern surface as a parameter. Although the finite thickness of the Al pattern (100 nm) together with the SiO_2_ underlayer and their effects on the electric field distributions should be taken into consideration, an outline of the electric field distribution around the gap is depicted: *E*_*z*_ values of different signs appear on both sides of the *E*_*z*_ = 0 zone at the gap centre, leading to an electric field variation that is much more gradual than the pattern shape but on the nanometre scale.

**Figure 3:**
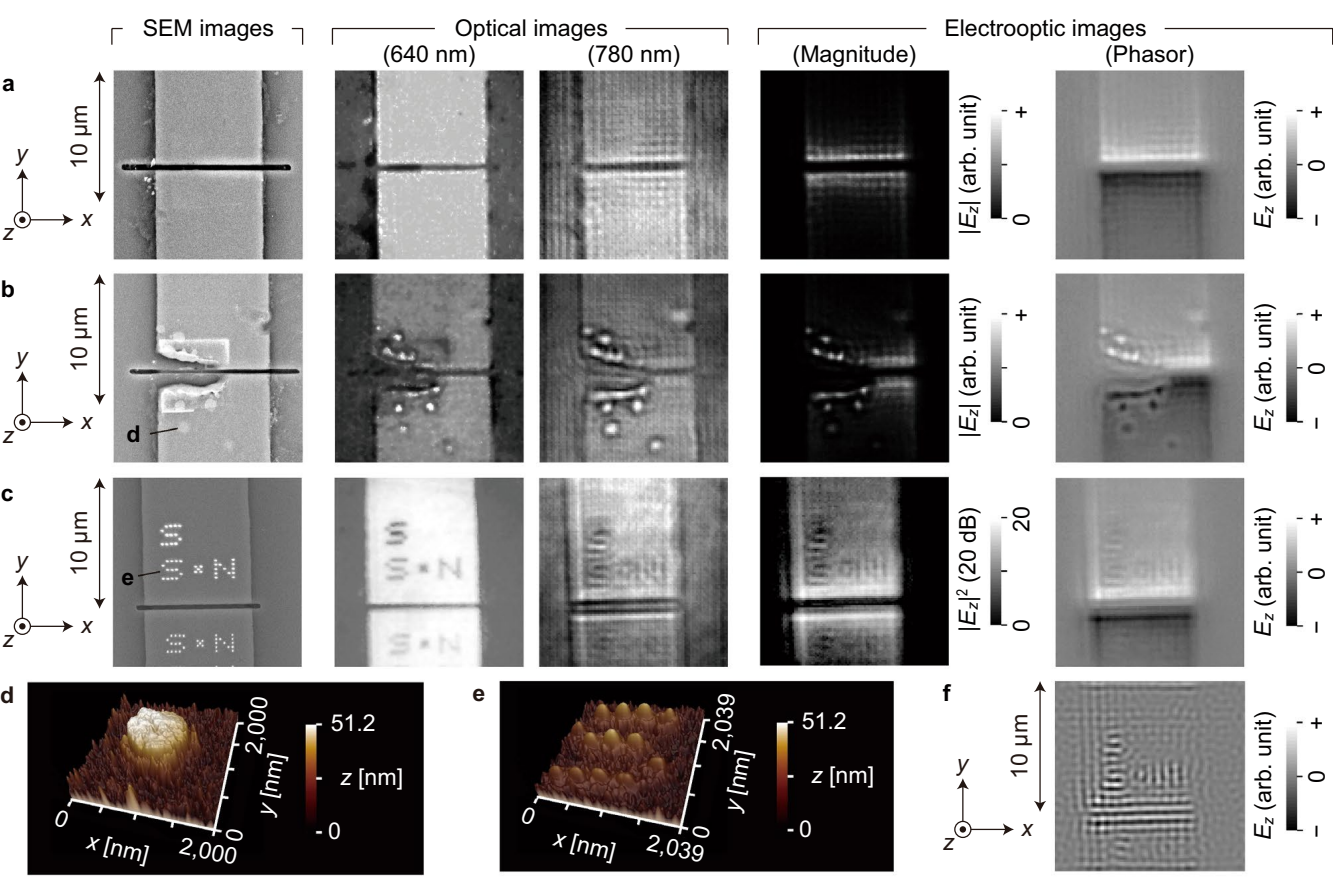
Electric field images for electrode patterns with nanometre-scale gaps. SEM and optical images are also included. (**a**) Simple 320 nm gap (pattern A). (b) Broken 270 nm gap (pattern B). (**c**) Gap with arrays of synthesized nanometre dots (pattern C). AFM images (**d**) and (**e**) are for the dots in (**b**) and (**c**), respectively. (**f**) Phasor image generated by the DEI process.

Images of three nanometre-scale gap patterns are shown in the leftmost column of Fig. 3a–c, which were taken by a scanning electron microscope (SEM, FEI Quanta FEG 250). Pattern A shown in Fig. 3a contains a simple line gap formed by the Ga focused ion beam (FIB) sputtering method, which appears as a dark line across the *y*-directional line pattern. The measured gap width is approximately 320 nm, while the line pattern is approximately 8.2 μm wide and narrower than the designed line pattern (10 μm) due to the side etching during the wet etching process.

Although pattern B shown in Fig. 3b was designed and processed in the same way as pattern A, its left half is broken, and nanometre-scale linear and dotted structures were formed accidentally, which are fortunately suitable for this work. One of the dotted Al structures is shown in Fig. 3d, which presents a three-dimensional (3D) image taken by an atomic force microscope (AFM, JEOL JSPM-5400) in tapping mode operation. The diameter of its circular skirt is approximately 1 μm, and its cone-shaped peak, somewhat shifted towards the gap, is approximately 50 nm high. The heights of other fine structures closer to the gap are approximately 100 nm. A 5.3 μm square region across the left half of the gap is recognized, which could be the cause of the gap destruction. Its speculated formation and gap destruction processes are described in the “Methods” section. The widths of the residual part of the gap and the line pattern are 0.27 μm and 8.56 μm, respectively.

For pattern C shown in Fig. 3c, arrays of Pt dots were artificially formed since nanometre-scale electric field concentrations at the abovementioned fine Al structures were observed, as described later. The Pt dots were laid out in alphabetic configurations (S and N) so that their electric field images could be easily determined, and the electric fields from adjacent dots worked as a resolution measure. The Pt dots were formed by a modified ion beam-assisted metalorganic chemical vapor deposition (IBA-MOCVD) method^18,19^, whose details are described in the “Methods” section. The Pt dots in the SEM image are bright, probably because of their high secondary electron emissivity. The contrast is different among the dotted characters since the dot shapes and sizes differ from character to character even though the dots were formed under the same conditions. The apparent deviation among the Pt dots is indicated in Fig. 3e, which presents a 3D AFM image for the second S-shaped dot array. The suspected origin of their deviation is described in the “Methods” section.

Two kinds of optical images are presented alongside the SEM images in Fig. 3a–c, which were taken by the optical system in Fig. 1a using the oil-immersion objective lens with the EO sensor in proximity to the nanometre-scale patterns. These images are basic references for the later-mentioned electric field images. The 640 nm optical images were taken in the same way as that in Fig. 2b and cut out. The 780 nm optical images were taken using the OLOS beam as an illuminator and the high-speed CMOS image sensor. Notably, their contrasts were maximized in their posttreatments, leading to their emphasized variations. Additionally, the vertical position of the patterned electrode sample was varied on a case-by-case basis to obtain the best focus for each optical image.

The 640 nm optical image in Fig. 3a shows that two Al electrode edges with a 320 nm gap in between are clearly separated, indicating that an optical nanometre-scale resolution is provided. However, the gap on the left half is wider than that on the right half, suggesting deviation from the ideal microscopic conditions. This is probably due to the pattern tilt caused by the curvature of the mound surface.

The dot arrays in the 640 nm optical image (Fig. 3c) appear as dark lines. This is probably because the optical reflection of Pt dots is lower than that of their surroundings and the optical resolution is lower than the dot pitch of approximately 320 nm. Furthermore, the focal plane is not at the dot summits but at the surface of the Al pattern. In contrast, the dot arrays in the 780 nm optical image are bright lines, which is probably due to the difference in the vertical positions of the sample.

### Image acquisition

Two kinds of ${E}_{z}$ movie snapshots are shown in part in the right columns of Fig. 3a–c. Here, a 2D distribution of the electric field amplitude is displayed in a magnitude image $\left|{E}_{z}(x,y)\right|$, while the distribution of the electric field phase $\varphi (x,y)$ is additionally displayed as a phasor image $\left|{E}_{z}(x,y)\right|\mathrm{cos}\varphi (x,y)$. Their image signals are sufficiently intense, suggesting that the present EO sensitivity is in a similar range to those with much thicker EO films. However, the enhancement of the electric field induced by the reduced gap width should be taken into consideration, and sensitivity calibration is needed for more quantitative discussion, which will be future work. In addition, the electric field images are well resolved, implying demonstration of a nanometre-scale resolution. Hereafter, their details are investigated with an emphasis on quantitative evaluation of the spatial resolution as well as the relationship between the electric field and optical images.

**Figure 4:**
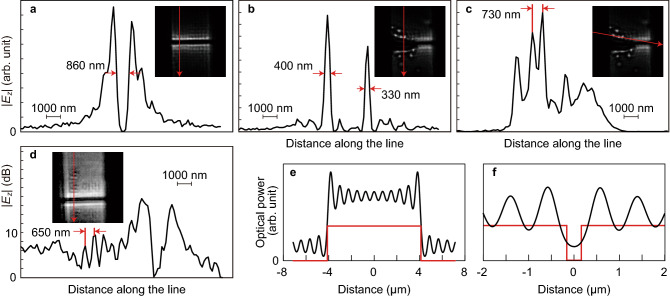
Line profiles of images for the electric field in the z direction *E*_*z*_. (**a**) Across the gap in pattern A. (**b**) Across the broken gap in pattern B. (**c**) Along the row of bright spots in pattern B. (**d**) Over the arrays of synthesized dots in pattern C. Optical power distributions at the DBR interface, simulated with the light reflected at the electrode patterns taken into account, across the electrode (**e**) and gap (**f**).

In the phasor image of Fig. 3a, *E*_*z*_ values of opposite signs convergent to the electrode edges appear on both sides of the gap, which is in good agreement with the abovementioned theoretical prediction. However, unexpected periodic intensity variations, whose periods are approximately five times the pixel pitch, appear therein due to the OLOS interference, which is discussed later. Figure 4a shows a profile of the electric field amplitude along the red line shown in the corresponding inset magnitude image. The dark line width was measured to be 860 nm; this is the narrowest width along the dark line, which has a varying width caused by the fabrication process, and could be determined not by the ultimate spatial resolution of the present LEI system but by the electric field distribution itself. Similar electric field distributions appear for the right half of the gap in Fig. 3b, where the dark line width was measured 710 nm along a line 3.88 μm away from the side edge. For the other half of the gap, electric fields convergent to the accidentally formed fine structures appear. An electric field profile along a line 5.19 μm from the side edge is shown in Fig. 4b as an example, where the measured widths for the bright dot and line are 400 and 330 nm, respectively. These could be regarded as response functions of the system to quasi-delta function inputs. Another line profile is shown in Fig. 4c, which contains two adjacent bright spots with a distance of 730 nm in between. Since the spatial resolution of an imaging system is generally defined as the shortest distance between two resolved adjacent spots in images, a nanometre-scale resolution is thus clearly demonstrated.

Most clearly imaged in Fig. 3c are the electric field distributions over the S-shaped array just above the gap, while those over the other two S arrays as well as N-shaped arrays are less apparent. The difference between S and N in the electric field image signal intensities can be ascribed to their different distances from the EO film caused by the tilt of the pattern surface (100 nm/8 μm). The electric field image divergence among the three S arrays comes from the variances in their distances from the gap, dot shapes, and distances between dots and the ZnTe film. An electric field line profile longitudinal to the S character is shown in Fig. 4d, which indicates 650 nm as the resolution of the three dotted lines in the S-shaped array.

The abovementioned LEI demonstrations can be summarized as follows. The spatial resolution of the present LEI system is in the nanometre-scale range and finer than 650 nm, as indicated in Fig. 4d. The dot pitches of approximately 320 nm for the third pattern were not resolved, which could be due to the poor electric field contrast caused by the nonoptimized focus and/or the lower proximity, although their details should be clarified in future work.

## Discussion

Hereafter, room to improve the spatial resolution is discussed. Factors regarding the optics are the OLOS wavelength, the focusing condition including the focusing tolerance, the NA value, and possible application of the super-resolution technique. A shorter wavelength leads to a higher optical spatial resolution. However, a high-speed optical modulator is less available, leading to a trade-off relation between the resolution and the bandwidth. In a low bandwidth case, a short-wavelength optical source modulated directly would be a solution. The focus of electric field images is highly delicate since electric field images are rather soft and ambiguous. Additional ways to optimize the focus are needed; for instance, marks on the EO film for measuring the focus could be effective. Furthermore, possible application of the confocal microscopy technique could suppress the vertical integration of the EO effect via reduced focusing tolerance. There is little room to improve the spatial resolution by increasing the NA of the oil-immersion objective lens as well as the illumination condenser lens. Super-resolution image creation methods are possibly applicable, including the detached EO imaging (DEI)^20^ technique described below.

For the EO sensor, further thinning of the two layers could improve the resolution. A thinner EO film could lead to resolution improvement, while unchanged EO sensitivity would be empirically expected since its thickness independence has been observed in resolution improvement trials^4,14^. The unclear reason for this phenomenon needs to be clarified in future work^21^. Although a thin EO film was provided by the polishing method in this work, other methods for EO film formation to obtain thinner EO films would be attractive. Thin film formation techniques such as crystal growth and polycrystal deposition are candidates. A thinner DBR stack could lead to improved proximity and resultant resolution improvement at the expense of interference pattern effect enhancement. Their trade-off relationship should be managed. The optimized material combination of a DBR stack, an optical source of lower coherence, and/or numerical calibration based on optical images could be effective for interference pattern effect suppression. Another area for improving the demonstrated resolution lies in the nanometre-scale electrode patterns, which have not been optimized. Their optimization would provide finer electric field distributions.

The abovementioned trade-off relationship in the design of the OLOS wavelength and the DBR stack is an issue to be solved together with the dust particle inclusion problem, which has been prevented to some extent by the mound structure introduction at the expense of extremely severe restrictions in universal LEI usage. Alternative methods for the prevention of dust particle inclusion should be developed to enable a wider variety of electrode pattern structures. LEI operation in a clean room could be a solution.

In the DEI framework, the *E*_*z*_ distribution at an electrode pattern surface can be inversely evaluated from the electric field distribution at an upper plane through the corresponding in-plane electric charge distribution on the electrode pattern. The details are described in the “Methods” section. The result of DEI application to the electric field image of the Pt dot array is shown in Fig. 3f, where the electrical length $\sum_{i}{\varepsilon }_{r}^{i}{t}_{i}=708 \mathrm{nm}$ induced by DBR layers was taken into consideration. Here, ${\varepsilon }_{r}^{i}$ and ${t}_{i}$ are the relative permittivity and thickness of the *i*th DBR layer, respectively. The contrast is enhanced, and the electric field distributions at the dot peaks are clearer; however, components of higher spatial frequencies are emphasized in the DEI numerical processing, suggesting the necessity of careful consideration as well as improvement of the method.

Finally, with a focus on the images in Fig. 3a, the effect of optical interference caused by the low reflectance of the EO sensor bottom coating is discussed. The interference pattern appears more significantly in the 780 nm image than in the 640 nm image, which could be due to the difference in their coherence lengths and the wavelength-dependent DBR reflectance. The coherence length of the present 640 nm LED is estimated to be 11 μm from its spectral half width at half maximum (18 nm), which is close to the electrode width and shorter than the *L*_*c*_ of the SLD (18 μm). The wavelength-dependent DBR reflectance is 0.55 at 640 nm and 0.47 at 780 nm.

To clarify the origin of the 780 nm interference pattern, a theoretical simulation for the optical power distribution at the EO sensor bottom coating was performed and its result is shown in Fig. 4e and f. The simulation was based on a simple 2D model with the following assumptions: the DBR thickness is zero, a quarter-wavelength space with a permittivity of 1 exists between the DBR layer and the Al electrode surface, multiple reflections are ignored, and incoherent return components whose optical path differences are longer than the *L*_*c*_ of the SLD are also ignored. The details are explained in the “Methods” section. The respective optical power profiles thus simulated at the ZnTe film bottom surface in the *x* and *y* directions are shown in Fig. 4e and f, which are in good agreement with the 780 nm optical image in Fig. 3a. This agreement suggests that the suspected origin is probable, although the experimental result contains a considerable amount of incoherent components. More specifically, the reflection at each DBR layer, multiple reflections, and components of longer path differences than the *L*_*c*_ of the SLD should be taken into account in the simulation model, which will be a future subject.

Here, we move to the interference pattern effect on electric field images. The interference pattern effect on electric field images is different from that on optical images since phase modulation is generated in the EO sensor, converted into intensity modulation and detected by the image sensor in the LEI optics, which should be taken into consideration. First, the interference pattern effect on the phase modulation is generally more gradual than that on the optical power distribution. Their difference can be explained more clearly by the following example: two optical beams with similar amplitudes and slightly different optical path lengths interfere. The optical amplitude is almost their sum, but the phase is their average. Second, the photodetected amplitude of the intensity modulation converted from the phase modulation is proportional to the instantaneous optical power at an image sensor pixel but is numerically normalized by the received optical power to eliminate the optical power nonuniformity. Therefore, the effect of the interfered optical power distribution is supposed to be suppressed if the optical detection is only for coherent components. However, the additional incoherent components are indeed high, and the numerical normalization effect disappears. Consequently, the interference affects the electric field images. Hence, taking the interference pattern effect into consideration is indispensable for more rigorous investigation of the electric field images in Fig. 3a–c and their line profiles in Fig. 4a–d. For instance, the contrast of electric field images could be enhanced by the interference pattern effect, resulting in a sharpening effect of electric field images and overestimation of the resolution. Fourier filters might be effective in suppressing the interference pattern effect if appropriate image processing could be provided through an understanding of the nature of the interference pattern effect.

A positive aspect of the interference pattern effect should be noted here. Since the interference patterns in Fig. 4e and f depend strongly on the distances between the DBR layers and the Al patterns, the distance could possibly be estimated from the patterns. To make the estimation more precise, the distribution and coherence of reflected light at the pattern surface need to be obtained. The former is possible by using an optical image without an EO sensor. The interference pattern effect could also be numerically removed from the electric field images if all the behaviours of the related light waves are comprehended.

## Concluding section

Real-time electric field imaging with a spatial resolution in the nanometre range has been successfully demonstrated by applying an oil-immersion objective lens and a highly thinned EO sensor to an LEI system. The acquired electric field images indicate a minimum linewidth of 330 nm and a minimum peak splitting of 650 nm, where the obtained best spatial resolution includes some uncertainties, but in the nanometric range, and room to improve the resolution has been suggested. Additionally, a few problems to be solved have been noted, including dust obstruction and the optical interference pattern effect.

## Methods

### LEI system

First, the operation principle of the present LEI system is briefly explained with an emphasis on the infinity correction polarization optical system within it. As shown in Fig. 1a, 780 nm light modulated at *f*_LO_ is launched from an OLOS source into the optics via a polarization-maintaining optical fibre (PMF) and a collimating lens, whose NA and focal distance (FD) are 0.19 and 60 mm, respectively. The NA is degraded to 0.11 by the narrow optical path thereafter. The launched LO light travels through a polarization beam splitter (PBS) to a ZnTe EO film placed either on a patterned electrode sample or within radio waves, where the EO effect occurs in accordance with the distribution of the evanescent or radio electric field at *f*_RF_, and the OLOS beam is overlaid with the distributed RF modulation. The 780 nm light returns to the PBS, and intensity modulation at an *f*_IF_ of 5 kHz is generated by optical intermixing. This frequency down-conversion process from *f*_RF_ to *f*_IF_ is spatially coherent within the laser beam cross section. The IF component travels through an imaging lens with an NA of 0.014 and an FD of 800 mm and is detected at a frequency *f*_IS_ (= 20 kHz ∼ 4*f*_IF_) in quadrature by each of the 100 × 100 pixels on a fast CMOS image sensor. Three kinds of electric field videos phase evolving at a visible frequency δ*f* (= *f*_IF_ − *f*_IS_/4), i.e., magnitude, phase, and phasor videos, are displayed in real time with an *f*_D_ of 10 frames per second typically. Related EO images at various phases and frequency bandwidths of the LEI technique can be found in the literature^1–4^. All the optics employed in the LEI system are applicable for both 780 nm and 640 nm except for the MZM device, the polarization controllers, the dichroic mirror and the DBR stack of the EO sensor.

Second, the present basis of the LEI technique is explained by the following specifications: the highest frequency of visualized electromagnetic waves, largest viewing area, highest optical magnification ratio, and maximum pixel number are 100 GHz^2^, 25 mm square^3^, 200^4^, and 256 × 256^22^, respectively. The dynamic range of the representative LEI system is 30 dB for an RF input of 5 dBm for the conventional electrode patterns. Additionally, unique LEI functionalities have been demonstrated such as wave vector mapping^23^ and application in high-definition wave imaging and high-frequency (HF) waveguide mode analysis^24^, visualization of rotating electric field vectors and their elliptical nature^25^, visualization and decomposition of Bloch states in metamaterial structures^26^, visualization of propagating waves in and around electromagnetic absorbers^27^, packet imaging^28^, and DEI^20^.

### EO sensor

The EO sensor in Fig. 1b consists of a glass ring, a glass plate, a glass block, and an EO film, all of which are adhesively assembled. The glass ring with a thickness of 2 mm is used to attach and detach the EO sensor to and from the EO sensor holder. The holder is designed so that the objective lens is sufficiently close to the glass plate and proximity is provided between the bottom of the EO film and the surface of a patterned electrode sample. The working distance, which is the distance between the lowest point of the objective lens and the upper surface of the glass plate, is 80 μm. The glass ring consists of two 1-mm-thick layers with two holes for an EO sensor handling tool. The glass plate with a thickness of 100 μm receives drops of the immersion oil. The glass block works as a base for the abrasive processing and coating of the EO film. The EO film is made of a (001) ZnTe thin crystal that is sensitive to the *z*-direction electric field *E*_*z*_ and is placed on 160 μm of glass beneath the oil. The upper and lower surfaces of the ZnTe film are coated by an AR coating on the glass side and an HR coating on the air side, respectively. The EO film is thinned by a polishing method provided commercially by Kogakugiken Corp.

The EO sensor holder is made of nylon containing chopped carbon fibres and was fabricated by a 3D printer. The bottom of the ZnTe film is set slightly below the bottom surface of the holder. The focal plane of the objective lens approximately coincides with the ZnTe film and is finely adjusted in the LEI observation.

### Patterned electrode samples

The electrode gaps and peripheral nanometre-scale structures, shown in Fig. 3, were prepared using an FIB machining apparatus (Japan Electro Optical Laboratory, JIB-4501Multi Beam System). The ion acceleration voltage and ion currents were 30 kV and 3 pA, respectively.

Nanometre-scale gaps were formed by the FIB sputtering method on Al electrodes of designed 10 μm width. For Pt dot formation, gasified trimethylcyclopentadienylplatinum was introduced over the focal spot of the Ga beam, which was irradiated for a short moment at room temperature. The deviations among the Pt dots in their shape and height probably come from nonuniform concentrations and/or flow rate of the MO gas as well as unstable astigmatism of the Ga ion beam. As shown in a 3D AFM image of the second S-shaped dot array (Fig. 3e), the dots are apparently ellipsoidal but fluctuate in their heights and shapes, supporting the abovementioned nonuniformity of Pt dots.

Regarding the origin of the broken gap in Fig. 3b, analyses using an energy dispersive X-ray spectrometer attached to the SEM machine indicated that the square is composed of Cr. A Cr layer of 50 nm thickness was indeed deposited before the FIB process to avoid the charging effect induced by ion beam irradiation and removed afterward by wet etching. The residual Cr square was probably formed by accidental deposition of mask material, for instance, carbon, on the Cr layer induced by unintentional electron or ion beam exposure in the initial phase of the FIB process. A current probably flows through this Cr square during the gap isolation test, leading to heat generation and resultant gap explosion.

### DEI

According to electromagnetism, the charge density $\rho \left(x,y\right)$ at a point P(*x*, *y*, 0) in a plane gives the static electric field vector **E**_0_ at a point Q(*u*, *v*, *h*) in an upper plane along the line PQ as

1 $${\mathbf{E}}_{0} \left( {u,v,x,y,z = h} \right) = \frac{{\rho \left( {x,y} \right)}}{{2\pi r^{2} \varepsilon_{0} \left( {1 + \varepsilon_{{\text{r}}} } \right)}}\left( {\frac{u - x}{r},\frac{v - y}{r},\frac{h}{r}} \right),\;\;\;\;\;\;\;\;\;r = \sqrt {\left( {u - x} \right)^{2} + \left( {v - y} \right)^{2} + h^{2} } ,$$

where ε_0_ is the permittivity of vacuum. The contribution of all of the charges in the patterned electrode plane is given by

2 $${\mathbf{E}}\left( {u,v,h} \right) = \int {\int\limits_{ - \infty }^{\infty } {dxdy{\mathbf{E}}_{0} \left( {u,v,x,y,z = h} \right),} }$$

whose *z* component is given by the following convolution:

3 $$E_{z} \left( {u,v,h} \right) = \left[ {\frac{{\rho \left( {x,y} \right)}}{{2\pi \varepsilon_{0} \left( {1 + \varepsilon_{{\text{r}}} } \right)}}} \right]*\left[ {\frac{h}{{\left( {x^{2} + y^{2} + h^{2} } \right)^{1.5} }}} \right] \equiv a*b.$$

Since the convolution of *a* and *b* corresponds to multiplication of their individual Fourier transforms (ℱ), $\rho \left( {x,y} \right)$ is eventually given by the following inverse Fourier transform (ℱ^−1^):

4 $$\rho \left( {x,y} \right) = 2\pi \varepsilon_{0} \left( {1 + \varepsilon_{{\text{r}}} } \right){\mathcal{F}}^{ - 1} \left( {\frac{{\varepsilon_{z} }}{B}} \right),$$

where ${\mathrm{\varepsilon }}_{z}\equiv \mathcal{F}\left[{E}_{z}\right]$ and $B\equiv \mathcal{F}[b]$. Application of Gauss’s flux theorem to ρ and *E*_*z*_ at the patterned electrode plane leads to

5 $$E_{z} \left( {x,y,0} \right) = \frac{{\rho \left( {x,y} \right)}}{{\varepsilon_{0} \left( {1 + \varepsilon_{{\text{r}}} } \right)}} = 2\pi {\mathcal{F}}^{ - 1} \left\{ {\frac{{{\mathcal{F}}\left[ {E_{z} \left( {x,y,h} \right)} \right]}}{B}} \right\},$$

suggesting that an *E*_*z*_ image at the patterned electrode plane is obtainable from an *E*_*z*_ image at the upper plane, which corresponds to the EO film plane in the LEI system.

### Interference model

The reflectivity of the Al pattern surface and other surfaces of the sample are assumed to be finite and zero, respectively, and the thickness of the EO film is assumed to be zero. These assumptions lead to the following equation expressing the OLOS amplitude on the EO film:

6 $$A\left( {x_{1} } \right) = B\left( {x_{1} } \right) + \mathop \smallint \limits_{{ - \frac{w}{2}}}^{\frac{w}{2}} C\left( {x,x_{1} } \right)\exp \left( {ikl\left( {x,x_{1} } \right) + i\pi + i\varphi \left( x \right) + i\varphi \left( {x_{1} } \right)} \right)dx,$$

Here, *x*_1_ and *x* are the positions on the EO film and on the pattern, respectively, while *w* is the pattern width. *B* and *C* are optical amplitudes at the EO film, which are reflected from the DBR layer and the Al pattern, respectively, and *A* is their sum. *k* is the wavenumber. φ and *l* are the *x*-dependent phase offset and optical path difference between *C* and *B*, respectively. The interference effect of the optical amplitudes is given by the square of the real part of the above equation, giving the optical power distribution ${P}_{\mathrm{LO}}({x}_{1})$ of the OLOS.

7 $$P_{{{\text{LO}}}} \left( {x_{1} } \right) = \left[ {B\left( {x_{1} } \right) + {\text{Re}}\left\{ {\mathop \smallint \limits_{{ - \frac{w}{2}}}^{\frac{w}{2}} C\left( {x,x_{1} } \right){\text{exp}}\left( {ikl\left( {x,x_{1} } \right) + i\alpha + i\varphi \left( x \right) + i\varphi \left( {x_{1} } \right)} \right)dx} \right\}} \right]^{2} = \left[ {B\left( {x_{1} } \right) + \mathop \smallint \limits_{{ - \frac{w}{2}}}^{\frac{w}{2}} \frac{{C\left( {x,x_{1} } \right)\cos \left( {kl\left( {x,x_{1} } \right) + \alpha + \varphi \left( x \right) + \varphi \left( {x_{1} } \right)} \right)dx}}{2}} \right]^{2} .$$

The optical power distribution in the *y*-direction is given in a similar way.
